# Correction to “Prevalence and Correlates of Distress Detected by the Distress Thermometer and Problem List in Lung Cancer Patients: A Cross‐Sectional Study”

**DOI:** 10.1111/1759-7714.70256

**Published:** 2026-02-20

X. Wei , J. Liu , H. Li , et al., “Prevalence and Correlates of Distress Detected by the Distress Thermometer and Problem List in Lung Cancer Patients: A Cross‐Sectional Study,” Thoracic Cancer 16, no. 23 (2025): e70200, 10.1111/1759-7714.70200.41392025 PMC12702687

The affiliation of the corresponding author, Xiaohong Ning, was incorrectly listed as “Department of Palliative Medicine, Tiantai County People's Hospital, Taizhou, China.”

The correct affiliation should read:

Department of Palliative Medicine, Peking Union Medical College Hospital, Chinese Academy of Medical Sciences & Peking Union Medical College, Beijing, China.

We apologize for this error.

